# Inflammation arising from obesity reduces taste bud abundance and inhibits renewal

**DOI:** 10.1371/journal.pbio.2001959

**Published:** 2018-03-20

**Authors:** Andrew Kaufman, Ezen Choo, Anna Koh, Robin Dando

**Affiliations:** 1 Department of Food Science, Cornell University, Ithaca, New York, United States of America; 2 Department of Molecular Medicine, College of Veterinary Medicine, Cornell University, Ithaca, New York, United States of America; New York University, United States of America

## Abstract

Despite evidence that the ability to taste is weakened by obesity and can be rescued with weight loss intervention, few studies have investigated the molecular effects of obesity on the taste system. Taste bud cells undergo continual turnover even in adulthood, exhibiting an average life span of only a few weeks, tightly controlled by a balance of proliferation and cell death. Recent data reveal that an acute inflammation event can alter this balance. We demonstrate that chronic low-grade inflammation brought on by obesity reduces the number of taste buds in gustatory tissues of mice—and is likely the cause of taste dysfunction seen in obese populations—by upsetting this balance of renewal and cell death.

## Author summary

Taste buds operate not only as sensors of essential nutrients but can also trigger powerful central reward from the consumption of hedonically pleasing food. Obese individuals have been reported to display a weakened sense of taste and thus may be driven to consume more calories to attain such reward. Here, we have analyzed the effects of obesity on taste buds and demonstrate that mice consuming a high-fat diet quickly become obese and display a pronounced loss of taste buds when compared to littermates sustained on a healthy diet. When the inflammatory response is impeded via genetic manipulation, we observe that mice no longer suffer taste loss, suggesting that taste dysfunction in obesity is a result of systemic inflammation. We also find that obesity-resistant mice consuming the same unhealthy diet have no change in the taste bud abundance, confirming that taste loss is a metabolic consequence of the obese state rather than a response to oral exposure to fat. Our results validate a role for taste in the genesis of obesity and suggest a novel direction in the treatment of obesity.

## Introduction

Obesity is one of the world’s most prevalent public health issues, affecting over one-third of United States citizens [1] and is associated with increased mortality, along with various comorbidities, including cardiovascular disease, diabetes, stroke, and cancer [2]. The treatment of obesity presents major challenges, including poor adherence to diets, with high rates of attrition [3]. Despite many studies outlining the effects of obesity, people continue to eat unhealthy diets. Various studies have investigated the relationship between body mass index (BMI) and taste perception with differing results. Earlier reports suggest little to no effect of BMI on taste sensitivity [4,5]. These studies tested detection thresholds for sweet stimuli in obese and normal-weight humans but failed to take into account individual variation in the perception of suprathreshold stimuli. With the invention of the generalized Labeled Magnitude Scale (gLMS), intensity ratings could be compared better across groups by anchoring the upper end of intensity scales to the “strongest imaginable sensation of any kind,” rather than strongest taste sensation, thus negating bias from the obese fundamentally perceiving taste differently [6]. Use of the gLMS revealed a negative association between BMI and perceived intensity of sweetness, umami, saltiness, and fatty taste [7–10]. This relationship, however, remains complex and methodology dependent, with not all studies in agreement and some studies finding an opposing relationship to be true [11]. Researchers have also explored the association between high BMI and decreased dopamine signaling from food intake, suggesting that the obese and overweight seek out more palatable foods to compensate for depressed reward [12,13]. Weight loss interventions, both gradual and acute (i.e., via bariatric surgery), have also proven to alleviate obesity-related alterations in taste function, suggesting a bidirectional relationship between adiposity and taste. Gastric bypass surgery in both rodents and humans can reestablish taste thresholds and reward signaling to levels seen in normal-weight controls, as well as decrease the preference for, and intake of, calorie-rich foods [14–21]. Despite evidence that taste is weakened in obesity, and rescued with weight loss intervention, few studies have investigated the molecular effects of obesity on the taste system. Despite this, various groups studying functional responses from taste buds of obese rodents have noted an altered response to sweet and fat stimuli [22–24], often accompanied by a decreased behavioral response.

A taste bud consists of a heterogeneous collection of 50 to 100 cells belonging to three functionally independent cell types. Type I cells are considered glial-like, provide structural support for the taste bud, and are likely responsible for salty taste detection [25]. Type II cells express G-protein–coupled receptors (GPCRs) for sweet, bitter, or umami tastants and transduce taste signals via the phospholipase C beta 2 (PLCβ2)/phosphatidylinositol bisphosphate (PIP2)/inositol triphosphate (IP3) cascade [26–28]. Type III cells form synapses with afferent nerve fibers and respond to sour taste [29,30]. Type II cells release ATP as their primary neurotransmitter [31,32], while Type III cells accumulate and release serotonin and synthesize the inhibitory transmitter gamma aminobutyric acid (GABA) [33]. Taste bud cells undergo continual turnover, even into adulthood, exhibiting an average life span of about 10 days [34]. Recently, a population of lingual stem cells has been identified that give rise to mature taste bud cells in the circumvallate papillae [35]. These cells express the leucine-rich repeat-containing G-protein–coupled receptor 5 (LGR5), which is also expressed by stem cells of the intestinal epithelium. LGR5 has been identified as a receptor for R-spondins, which activate the Wnt signaling pathway [35,36]. Upon activation of Wnt, these LGR5-expressing stem cells differentiate into immature sex-determining region Y box 2 (SOX2^+^) cells and migrate into the taste bud, losing their LGR5 expression in the process. SOX2 is required for the differentiation of endodermal progenitor cells of the tongue into mature taste bud cells and represents one of the last steps before terminal differentiation [37].

There are a number of processes that affect the generation of new functional taste cells from immature progenitor cells. Acute lipopolysaccharide (LPS)-induced inflammation has been shown to inhibit proliferation of taste progenitor cells and reduce the number of newly born cells entering taste buds. Furthermore, LPS-induced inflammation was shown to moderately shorten the average lifespan of mature taste bud cells [38], alongside a notable immune response. These experiments show the effects of inflammation in short-term, acute administration, a relatively rare occurrence. More common in human populations is the chronic and systemic low-grade inflammation found in obesity, the effects of which are yet to be fully catalogued in taste. While an acute immune response fundamentally differs from the sterile low-grade immune activity observed with obesity, notable parallels still persist. Evidence indicates that a state of low-grade chronic inflammation has a crucial role in the pathogenesis of obesity-related metabolic dysfunction [39,40]. With increased amounts of visceral adipose tissue, the production of proinflammatory cytokines—including tumor necrosis factor α (TNFα), interleukin 6 (IL6), and C-C motif chemokine ligand 2 (CCL2)—is up-regulated in the obese, with the potential to act on receptors within the taste bud, inducing cell death cascades [41,42]. TNFα in particular has been well documented as a key mediator of obesity-related pathologies [39,40,43]. TNFα-null mice have notably superior insulin sensitivity when obese through diet, with lower free–fatty acid levels [44] and with triglycerides and leptin also reduced [45]. This study investigates the effects of low-grade inflammation on the regenerative processes of taste buds in the obese state, demonstrating a biological mechanism for taste dysfunction seen in human sensory studies of obese populations.

## Results and discussion

### High-fat diet induces obesity and increases TNFα in taste

**Fig 1:**
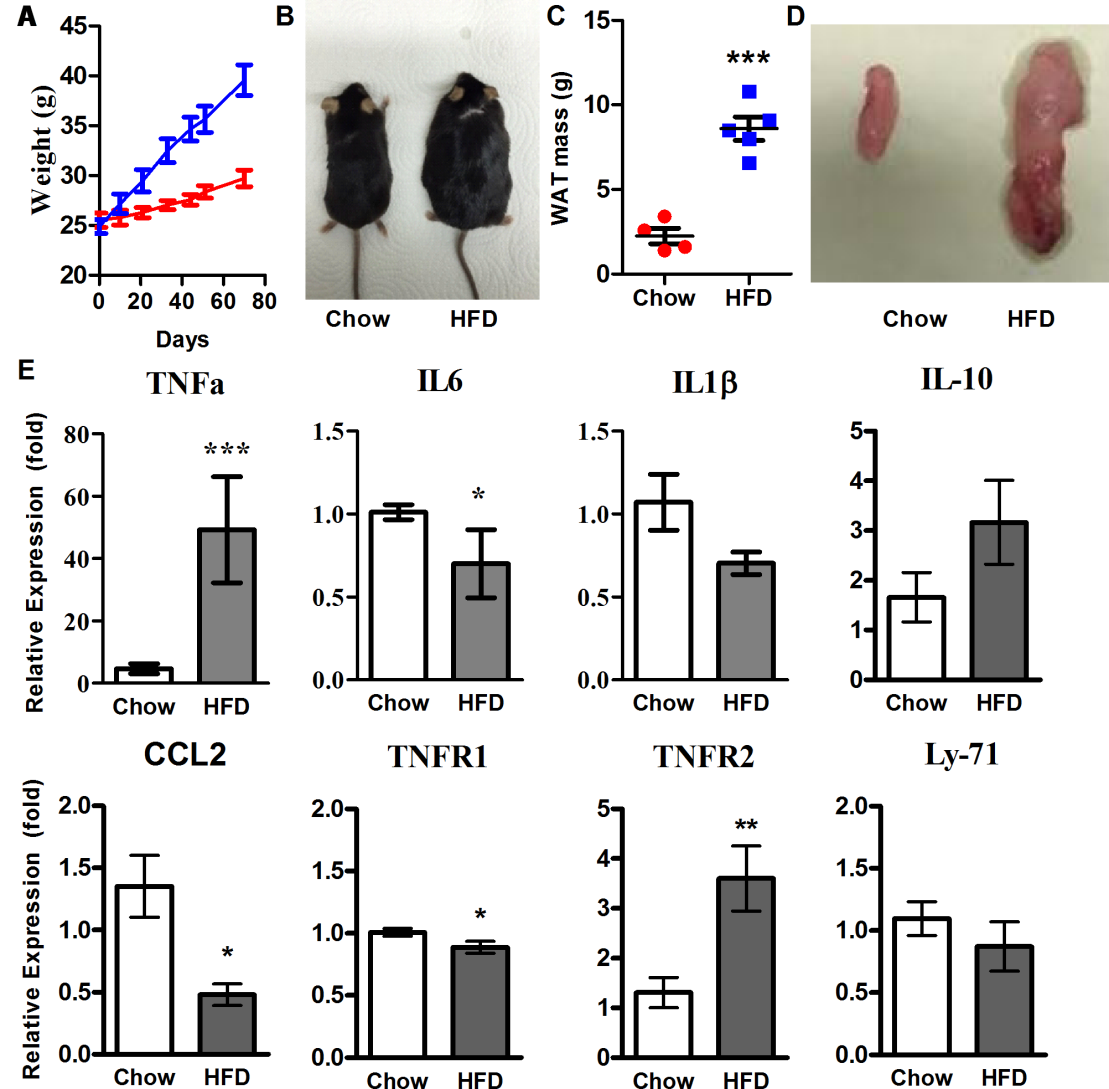
HFD induces adipogenesis and increases expression of TNFα. (A) Growth curve of C57Bl/6 WT male mice over 8-week ad libitum feeding with standard chow (red) versus HFD (blue). (B) Representative image of chow- and HFD-fed WT male mice after 8 weeks. (C) WAT mass from WT male mice after 8-week treatment period of standard chow and HFD (*n* = 4, 5, respectively). (D) Representative image of epididymal fat pads from chow- and HFD-fed WT male mice after 8 weeks on respective diets. (E) qRT-PCR analysis of the expression of inflammatory markers in CV papillae after 8-week treatment with chow or HFD (*n* = 4–6 each). Relative gene expression levels are shown (fold change). β-actin was used as the endogenous control gene for relative quantification. Error bars represent SEM. * = *p <* 0.05; ** = *p <* 0.01; *** = *p <* 0.005. Underlying data can be found in S1 Data. CCL2, C-C motif chemokine ligand 2; CV, circumvallate; HFD, high-fat diet; IL1β, interleukin 1 beta; IL6, interleukin 6; IL10, interleukin 10; Ly-71, lymphocyte antigen 71; qRT-PCR, quantitative real-time reverse transcription polymerase chain reaction; SEM, standard error of the mean; TNFα, tumor necrosis factor α; TNFR1, tumor necrosis factor receptor 1; TNFR2, tumor necrosis factor receptor 1; WAT, white adipose tissue; WT, wild-type.

Obesity is associated with a number of comorbidities and pathologies, with many stemming from the increase in circulating proinflammatory cytokines [39,40]. We split littermate 8-week-old wild-type C57Bl/6 male mice, consuming either a standard chow or a high-fat diet (HFD) for a period of 8 weeks (Fig 1A), into 2 cohorts. The HFD consisted of 58.4% fat to the chow’s 14% (see Materials and methods for full details). Mice gained significantly more weight when fed the HFD than those fed standard chow (Fig 1A and 1B), and postmortem analysis showed that a large amount of weight gained by the HFD-fed mice was in the form of white adipose tissue (Fig 1C and 1D).

We then tested whether HFD was sufficient to induce an inflammatory response associated with increased adiposity. The expression of several inflammatory markers in circumvallate papillae taste buds was examined. Acute induction of systemic inflammation via intraperitoneal injection of LPS has previously been demonstrated to shorten the lifespan of adult taste bud cells and reduce the population of supporting progenitor cells [38]. There are differences in immune response evident between the results of this study and our own, due to this study being concerned with an acute inflammatory response and not a low-grade persistent inflammatory response like in our study, where a differing immune response would be expected. Quantitative real-time reverse transcription polymerase chain reaction (qRT-PCR) analysis showed that, after only 8 weeks, expression of TNFα in taste tissues of HFD-fed mice was significantly increased, compared to lean controls (Fig 1E), with only moderate variation in interleukins at this time point. This was supported by concurrent altered expression of TNF receptors 1 and 2. Although similar mRNA expression patterns were appreciated in nontaste versus taste tissues for TNF receptors, minimal changes were observed for other markers (S1 Fig). These results suggest that prolonged obesity can induce a robust yet specific inflammatory response in the taste epithelium, consistent with reports localizing components of the inflammatory response to taste buds [42].

### Fewer taste buds in obese mice

**Fig 2:**
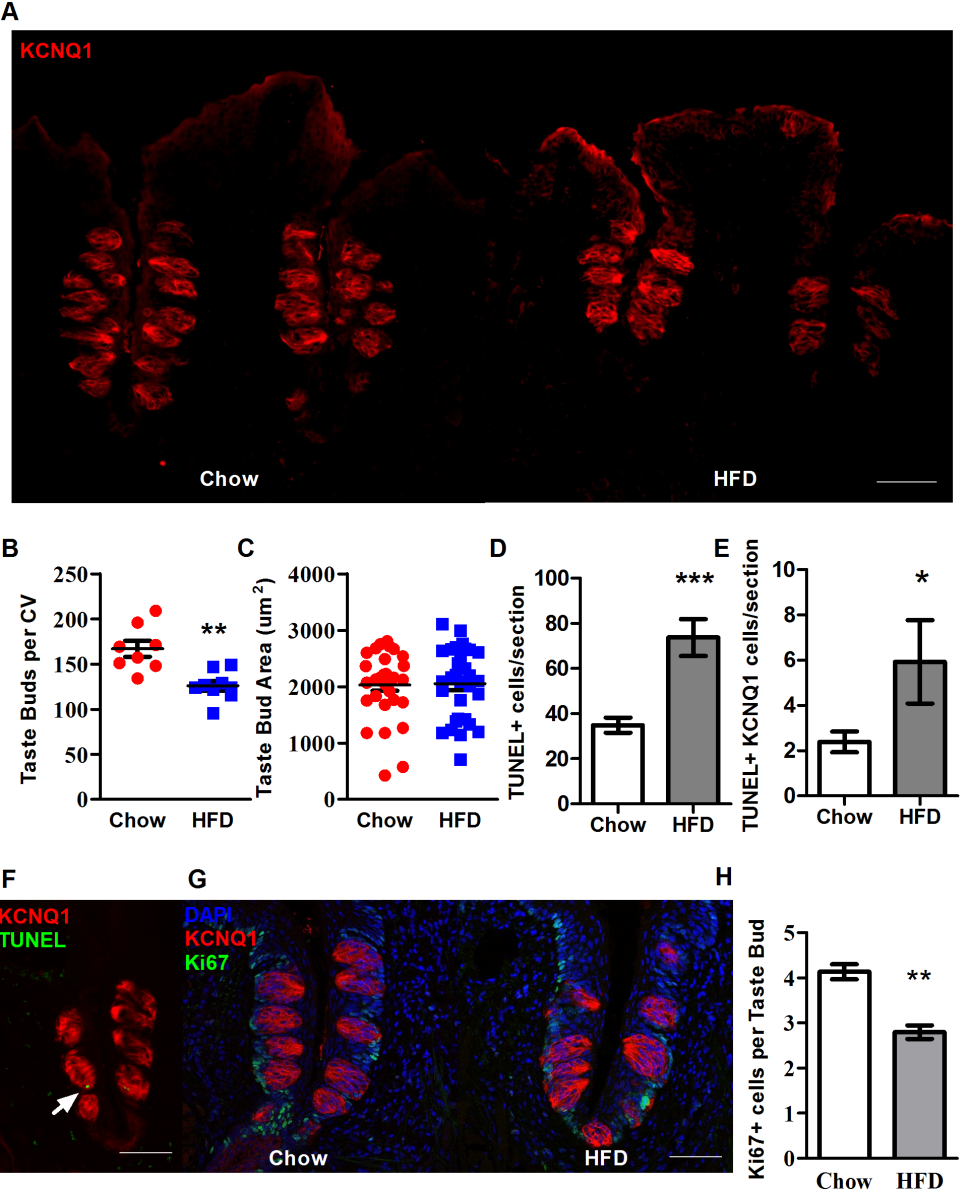
Loss of taste buds and taste transduction machinery in obese WT mice. (A) Fluorescent images of CV papillae sections stained with antibody against KCNQ1. Scale bars, 50 μm. (B) Total numbers of taste buds in CV papillae of chow- and HFD-fed WT male mice after 8 weeks shows a reduction in taste bud abundance in obese mice (*n* = 8, 9). (C) Taste bud area from chow- and HFD-fed WT male mice after 8 weeks reveals no change (n = 31, 32, respectively). (D) Counting of TUNEL-positive cells in CV papillae of chow- and HFD-fed mice (*n* = 23, 14 sections). (E) Counting of TUNEL-positive cells also expressing KCNQ1 in chow- and HFD-fed mice. (F) Representative image of TUNEL staining (green, at arrow) of KCNQ1-reactive taste cells (red). (G) Confocal fluorescent images of CV papillae stained with antibodies against KCNQ1 (red) and Ki67 (green) from chow- and HFD-fed WT male mice after 8 weeks, with DAPI staining for nuclei (blue). (H) Quantification of Ki67-labeled cells per taste bud in CV epithelium. Only the Ki67-labeled cells that were in direct contact with taste buds were counted (*n* = 3 each). Error bars represent SEM. * = *p <* 0.05; ** = *p <* 0.01; *** = *p <* 0.005. Underlying data can be found in S1 Data. CV, circumvallate; HFD, high-fat diet; KCNQ1, potassium voltage-gated channel subfamily Q member 1; Ki67, antigen KI-67; SEM, standard error of the mean; WT, wild-type.

Correlations have previously been reported between taste bud density and perceived taste intensity [46]. It is not clear whether human taste bud density changes longitudinally outside of a decline with aging; however, those with more taste buds seem to perceive taste stimuli as more intense. Human taste function has also been noted to decline with weight gain in college-age males [47]. In order to examine taste deficiency with obesity, we first measured the abundance of taste buds in obese mice compared to lean controls. Following the same dietary treatments described above, tissue sections containing circumvallate papillae were processed for immunofluorescent staining using antibodies against potassium voltage-gated channel subfamily Q member 1 (KCNQ1), a marker for taste bud cells [48]. Representative immunostaining images from lean and obese mice are displayed in Fig 2A, with quantification of total taste bud counts for whole circumvallate papillae revealing a significant reduction in taste bud abundance in obese mice (Fig 2B). The abundance of taste buds in obese mice significantly declined but with no difference in the size of taste buds evident between lean and obese cohorts (Fig 2C). Parenthetically, no change in the balance between Type I, II, and III cells was observed between chow- and HFD-fed mice (S1B Fig).

These data support the hypothesis that taste dysfunction in the obese may originate from a fundamental change in gustatory morphology, arising from a reduction in taste bud abundance. In addressing a mechanism, a rundown of taste cells might reasonably be ascribed to an increase in apoptosis of taste cells. Apoptosis is classically assayed with TUNEL staining; however, TUNEL-positive cells tend to be very rare in taste buds, as observed in rats [49], in mice [50,51], and in guinea pigs [52], with Nguyen et al. [53] recording less than 1% of cells in healthy control mice taking on TUNEL labeling and Takeda et al. [50] noting that epithelial cells took on TUNEL staining more readily than taste bud cells. In our testing (Fig 2D, 2E and 2F), TUNEL staining was significantly higher in CV papillae sections as a whole (*p* < 0.001), in addition to being higher in taste cells (cells positive for both TUNEL and KCNQ1 staining, *p* = 0.0457), although there were low overall numbers of TUNEL-positive cells in taste, in agreement with observations from other groups [49–53].

### Taste stem and progenitor cell loss in obesity

Studies suggest that taste bud cells, as well as surrounding nontaste epithelial cells, are derived from a pool of multipotent progenitor cells that exist in the basal regions surrounding taste buds [35,36,54,55]. To investigate whether diet-induced obesity suppresses proliferation of taste bud progenitor cells, we performed immunostaining using antibodies against antigen KI-67 (Ki67)—an important marker for actively proliferating cells in the lingual epithelium [38] (Fig 2G)—highlighting cells located in the basal periphery of adult taste buds, considered a niche for taste progenitor cells. Obese mice exhibited fewer cells immunoreactive for Ki67. Sections were also stained for KCNQ1 to reveal the location of taste buds, with only Ki67-immunoreactive cells in contact with taste buds counted in quantification [38] (Fig 2H), also revealing a decrement in the obese mice. A loss of input from taste buds would likely encourage overconsumption to compensate for this depressed input, as has been observed when blocking input from sweet taste receptors in a human population, resulting in a drive for more highly sweetened (and thus higher-calorie) stimuli [56].

### Deletion of TNFα protects taste buds from detrimental effects of obesity

**Fig 3:**
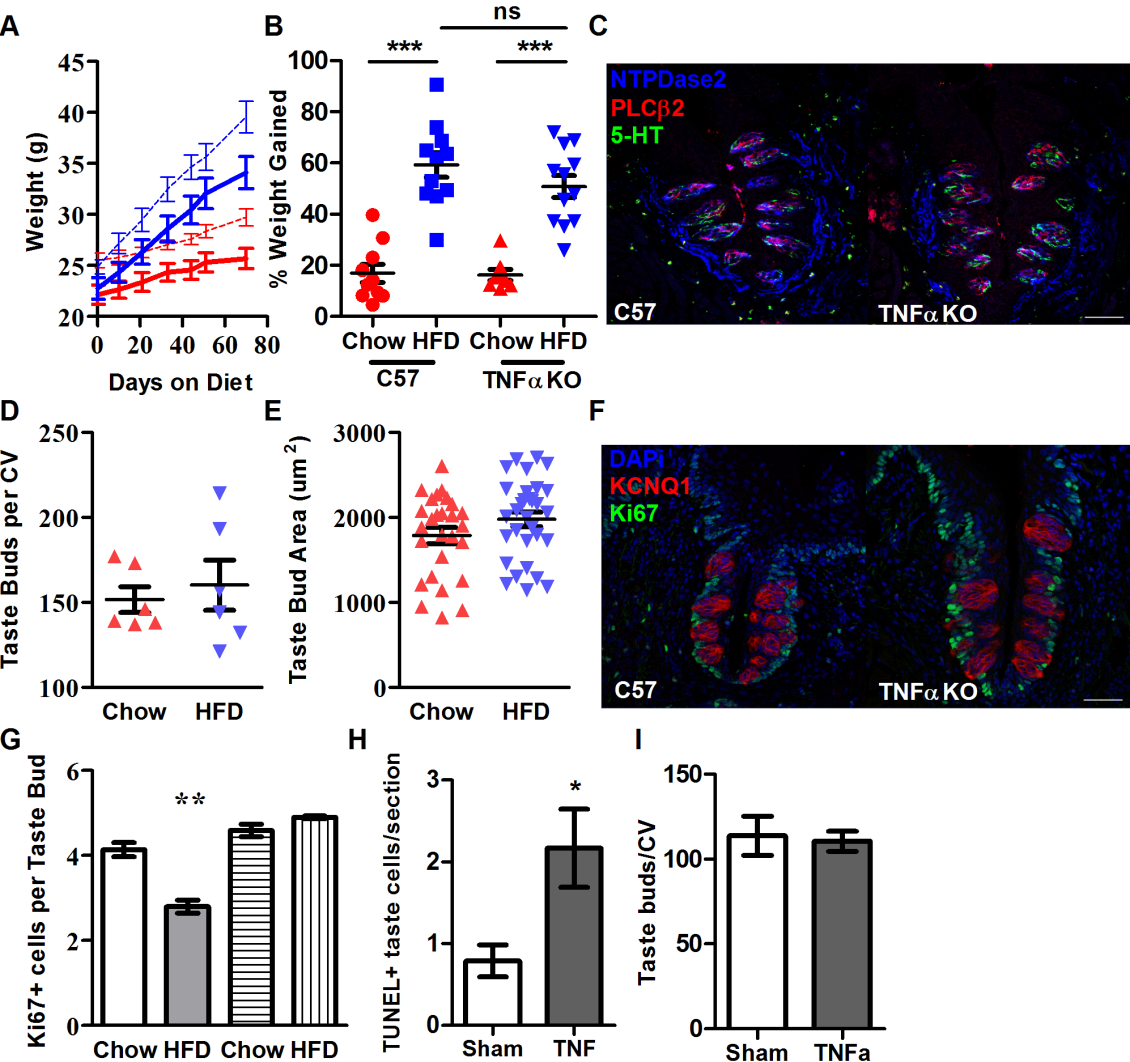
HFD induces adipogenesis in male TNFαKO mice similarly to WT mice but does not cause loss of taste buds or impede proliferative capacity of taste tissues. (A) Growth curve of TNFαKO versus WT mice over 8-week ad libitum feeding with standard chow and HFD. TNFαKO mice shown in solid lines, C57 dashed; chow-fed mice in red, HFD in blue (*n* = 8–12). (B) Percentage weight gained by TNFαKO versus WT mice after 8-week treatment period of standard chow and HFD. (C) Fluorescent images of CV papillae stained with antibodies against NTPDase2 (blue), PLCβ2 (red), and 5-HT (green) from chow- and HFD-fed TNFαKO mice after 8 weeks. Scale bars, 50 μm. (D) Total number of taste buds counted from CV papillae of chow- and HFD-fed TNFαKO mice after 8 weeks (*n* = 6). (E) Taste bud area measured from chow- and HFD-fed TNFαKO mice after 8 weeks on respective diets (*n* = 27, 31). (F) Fluorescent images of CV papillae stained with antibodies against KCNQ1 (red) and Ki67 (green) from chow- and HFD-fed TNFαKO mice after 8 weeks. DAPI shown in blue. (G) Number of Ki67-labeled cells per taste bud in CV epithelium of WT versus TNFαKO mice, on chow and HFD. Only the Ki67-labeled cells that were adjacent to taste buds were counted. Obesity reduced the number of cells labeled with Ki67 antibody in WT but not TNFαKO mice (*n* = 3 each). (H) Number of cells undergoing apoptosis is greater in mice injected with TNFα than with vehicle (sterile PBS, *n* = 4 mice in each group). (I) WT mice injected lingually with TNFα do not, however, lose taste buds after a single injection (*n* = 4). Error bars represent SEM. * = *p <* 0.05; ** = *p <* 0.01; *** = *p <* 0.005. Scale bars, 50 μm. Underlying data can be found in S1 Data. 5-HT, serotonin; CV, circumvallate; HFD, high-fat diet; KCNQ1, potassium voltage-gated channel subfamily Q member 1; Ki67, Antigen KI-67; KO, knockout; NTPDase2, ectonucleoside triphosphate diphosphohydrolase 2; PBS, phosphate buffered saline; PLCβ2, phospholipase C beta 2; SEM, standard error of the mean; TNFα, tumor necrosis factor α; WT, wild-type.

TNFα is linked with a number of negative outcomes related to obesity, including insulin resistance [57] and the induction of further inflammation [58]. TNFα expression is highly up-regulated in the taste tissues of obese wild-type mice, compared to lean controls (Fig 1E). In order to determine if TNFα was necessary to cause the dysfunction in taste bud renewal that obese mice exhibit, we employed the same dietary treatment previously described to 2 cohorts of 8-week-old B6.129S-Tnf^tm1Gkl^/J male mice. These TNFα-null mice are marginally smaller in size than wild-type mice at sexual maturity but gained a similar percentage of their body weight on either diet when compared to wild-type mice (Fig 3A and 3B), suggesting that deletion of TNFα did not impede adipogenesis resulting from HFD feeding.

Immunostaining and taste bud counting from circumvallate papillae sections of lean and obese TNFα-null mice did not reveal a reduction in taste bud abundance with obesity as that seen in wild-type mice (Fig 3C and 3D), although the absolute number of taste buds was slightly (though not significantly) smaller than C57s, likely a reflection of their smaller body size. Taste bud area was also unaffected by diet in TNFα-null mice (Fig 3E).

Similar results were observed following immunostaining for Ki67 in TNFα-null mice. Obese mice did not experience the reduction in Ki67^+^ taste progenitor cells demonstrated in obese wild-type mice (Fig 3F and 3G). Similar to overall taste bud count, the number of Ki67^+^ cells per taste bud was slightly (although not significantly) higher in TNFα-null mice than controls (Fig 3G), indicating no discernable damage to the proliferative capacity of taste buds in obese mice lacking TNFα.

Extrinsic apoptosis can be powerfully triggered by TNFα. Directly injecting TNFα into the tongues of healthy lean mice led to an increase in apoptosis in taste buds, with more than double the number of cells expressing KCNQ1 positive for TUNEL staining after 1 week in TNFα-injected mice compared to those injected with vehicle (Fig 3H). The absolute number of taste buds in these mice did not change with this single TNFα dose (Fig 3I), likely reflective of the acute, rather than chronic, TNFα exposure. To determine whether a reduction in taste buds, proliferative capacity, or the inflammatory response itself stemmed from the adiposity of the animals—or instead from the chronic oral exposure to a HFD—gene expression analysis and immunostaining experiments were also performed in an obesity-resistant transgenic mouse model. Adipocyte-specific deletion of Sel1L from mice results in inefficient metabolism of fatty acids and significantly reduced adiposity when maintained on a HFD [59]. These animals were subjected to the same dietary treatments as wild-type mice and ate a similar amount of chow or HFD to wild-type mice but did not show an increase in expression of TNFα after 8 weeks. Furthermore, expression of taste bud machinery and markers of self-renewal were similarly unaffected in these mice (S2 Fig). Representative images of circumvallate papillae from chow- and HFD-fed Adipo-Sel1L knockout mice immunostained with antibodies against ectonucleoside triphosphate diphosphohydrolase 2 (NTPDase2), PLCβ2, and serotonin (5-HT) to reveal mature Type I, Type II, and Type III taste cells, respectively, also showed no difference between dietary treatments, as with TNFα-null mice. This would imply that the metabolic effects of obesity trigger a decline in taste and not merely the oral exposure to fat.

These data together suggest that gross adiposity stemming from chronic exposure to a HFD is associated with a low-grade inflammatory response, causing a disruption in the homeostatic mechanisms of taste bud maintenance and renewal. Inhibition of proinflammatory cascades, whether by genetic deletion of TNFα or by resistance to adipogenesis, is sufficient to avert the systematic loss of taste buds due to an obesogenic diet and may highlight novel therapeutic targets in the alleviation of taste dysfunction in obese populations.

## Materials and methods

### Ethics statement

Studies were performed according to protocol 2012–0080 approved by the Cornell University Institutional Animal Care and Use Committee. Mice were euthanized with CO_2_ followed by cervical dislocation.

### Animals

C57BL/6 and B6.129S-Tnf^tm1Gkl^/J mice were purchased from Jackson Laboratory (Bar Harbor, ME). Adipocyte-specific Sel1L^-/-^ (AKO) mice were a gift from Dr. Ling Qi of the University of Michigan Medical School. Mice were housed in a climate-controlled environment at the East Campus Research Facility at Cornell University College of Veterinary Medicine. Eight-week old male mice were fed on either standard rodent diet consisting of 14% fat, 54% carbohydrate, and 32% protein (Harlan Teklad 8604) or a HFD consisting of 58.4% fat, 26.6% carbohydrate, and 15% protein (Harlan Teklad TD.03584) for a period of 8 weeks. Inguinal and epididymal white adipose tissue deposits were excised and weighed post mortem.

### qRT-PCR

Following dietary treatment, mice were euthanized, and tongues were incubated in ice-cold normal Tyrode’s solution (NaCl 135 mM, KCl 5 mM, CaCl_2_ 2 mM, MgCl 1 mM, NaHCO_3_ 5 mM, HEPES 10 mM, Glucose 10 mM, Sodium Pyruvate 10 mM, pH 7.4). The excised tongue was injected subepithelially with a mixture of Dispase II (2.5 mg/ml), Collagenase A (1 mg/ml), Elastase (0.25 mg/ml), and DNaseI (0.5 mg/ml) in Tyrode’s solution and incubated at room temperature for 20 minutes. Epithelial sections containing taste buds and nontaste epithelial control tissues were collected [60] and total RNA extracted using Absolutely RNA Microprep Kit (Stratagene, Cedar Creek, TX). Samples were reverse-transcribed into cDNA using qScript cDNA Supermix (Quanta Bio, Beverly, MA) and diluted using standard curves. qRT-PCR using Power SYBR Green PCR Master Mix (Applied Biosystems, Foster City, CA) was run on a QuantStudio 6 Flex Real-Time PCR System (Thermo, Waltham, MA). Relative quantification was performed using QuantStudio PCR Software, based on the 2^-ΔΔCt^ method. β-Actin was used as a housekeeping control gene. All genes were tested in triplicate, with samples sizes reflecting the number of animals tested.

### Taste bud counting and size measurement

Mice were euthanized and tongues removed and fixed in 4% PFA/PBS solution. Tissues were cryosectioned at 10 um thickness and washed in PBS. CV sections were incubated in blocking buffer (2% bovine serum albumin, 0.3% Triton X-100, 2% donkey serum) at room temperature for 2 hours. The sections were then incubated in polyclonal goat antibody against KCNQ1 at 4°C overnight. The sections were washed 3X in PBS and further incubated in AlexaFluor 647-conjugated donkey antigoat secondary antibody at room temperature for 2 hours. Sections were washed 3 times in PBS and mounted with ProLong Gold mounting medium containing DAPI stain. Images were taken using an Olympus IX71 Inverted Fluorescent Microscope. Taste buds were visualized with ImageJ (NIH, Bethesda, MD) and counted from every fifth section to avoid double counting. To calculate taste bud size, the perimeter of the largest taste bud (from every fifth section) was outlined, and the corresponding area was calculated with ImageJ.

### Taste bud cell type staining

Mice were euthanized and tongues removed and fixed in 4% PFA/PBS solution. Tissues were cryosectioned at 10 um thickness and washed in PBS. CV sections were incubated in blocking buffer (2% bovine serum albumin, 0.3% Triton X-100, 2% donkey serum) at room temperature for 2 hours. The sections were then incubated in a mixture of polyclonal goat antibody against PLCβ2 (Santa Cruz #sc-31759, 1:1000), rat polyclonal antibody against 5-HT (Millipore #MAB352, 1:1000), and rabbit monoclonal antibody against NTPDase2 (Sevigny lab, CHU de Québec, #mN2-36I6, 1:500) at 4°C overnight. The sections were washed 3 times in PBS and further incubated in AlexaFluor 647-conjugated donkey antigoat secondary antibody at room temperature for 2 hours. Sections were washed 3 times in PBS and mounted with Prolong Gold medium containing DAPI stain. Images of every fifth section were taken using a Zeiss LSM710 confocal microscope, with 1 taste bud per section selected with a random-number generator, taste buds numbered in epithelia from left to right, and cells from each cell type counted.

### Ki67 immunostaining and cell counting

Mice were euthanized and tongues removed and fixed in 4% PFA/PBS solution. Tissues were cryosectioned at 10 um thickness and washed in PBS. CV sections were washed in methanol plus 3% hydrogen peroxide for 30 minutes and then rinsed 3 times in PBS. Sections were incubated in blocking solution (2% bovine serum albumin, 0.3% Triton X-100, 2% donkey serum) for 2 hours and then further incubated overnight at 4°C with antibodies against KCNQ1 (Santa Cruz #sc-10646, 1:1000) and Ki67 (Thermo Scientific #RM-9106-S1, 1:200). After washing 3 times with PBS, the sections were incubated with AlexaFluor 647-conjugated antigoat secondary (for KCNQ1) and AlexaFluor 488-conjugated antirabbit secondary (for Ki67) at room temperature for 2 hours. Sections were washed and mounted with ProLong Gold mounting media. Images of every fifth section were taken with a Zeiss LSM710 confocal microscope. Only Ki67-labeled cells immediately surrounding a taste bud (defined by the KCNQ1 staining) were counted.

### TNFα lingual injections

Healthy lean mice at weeks 15 to 16 were injected in the anterior tongue with 200 ng of mouse TNFα in 10 uL of sterile PBS. Injections were with an insulin syringe and took place under isofluorane. Mice were under anesthesia for under 5 minutes. Controls underwent the same procedure, injected with only 10 uL sterile PBS. Mice were monitored post injection and feeding tracked for several days to ensure no change in intake. One week later, mice were euthanized and taste tissues collected for sectioning as above.

### TUNEL staining

Sections were sectioned at 10 um, as in staining protocols above. TUNEL staining used the Promega DeadEnd Fluorometric TUNEL kit, with which cells were briefly permeabilized using 5% Triton X-100 in PBS; washed in PBS; equilibrated in 100 μl eq. buffer at room temperature for 10 minutes; labelled with TdT mix for 50 minutes at 37°C shielded from light—the reaction stopped with 2-time SSC (15 minutes)—washed 3 times in PBS/0.3% Triton/2% BSA; and finally washed in PBS (5 minutes each). Slides were then blocked, and immunostained, as above.

## Supporting information

S1 Fig Non-taste tissues from lingual epithelium experience a less severe inflammatory response with obesity. (A) qRT-PCR of nontaste epithelium reveals the majority of immune response from obesity arises from taste buds themselves (*n* = 5 each). Relative gene expression levels are shown (fold change). β-actin was used as the endogenous control gene for relative quantification. * = *p <* 0.05; ** = *p <* 0.01; *** = *p <* 0.005. Underlying data can be found in S1 Data. qRT-PCR, quantitative real-time reverse transcription polymerase chain reaction.
(TIF)

S2 Fig Obesity-resistant mice show minimal changes to taste with HFD. (A) Minimal change to markers of taste, cell lineage, or apoptosis in mice resistant to obesity. Relative gene expression levels are shown (fold change). β-actin was used as the endogenous control gene for relative quantification. (B) Representative images revealing no taste bud loss in obesity-resistant mice. Error bars represent SEM. * = *p <* 0.05; ** = *p <* 0.01; *** = *p <* 0.005. Scale bars, 50 μm. Underlying data can be found in S1 Data. SEM, standard error of the mean.
(TIF)

S1 Data Supporting data. (XLSX)

S1 Table qRT-PCR primer sequences. (DOCX)

## Abbreviations

5-HT: serotonin
AKO: adipocyte-specific SEl1L^-/-^
BMI: body mass index
CCL2: C-C motif chemokine ligand 2
GABA: gamma aminobutyric acid
gLMS: generalized Labeled Magnitude Scale
GPCR: G-protein–coupled receptor
HFD: high-fat diet
IL6: interleukin 6
IP3: inositol triphosphate
KCNQ1: potassium voltage-gated channel subfamily Q member 1
Ki67: antigen KI-67
LGR5: leucine-rich repeat-containing G-protein–coupled receptor 5
LPS: lipopolysaccharide
Ly-71: lymphocyte antigen 71
NTPDase2: ectonucleoside triphosphate diphosphohydrolase 2
PBS: phosphate buffered saline
PCR: polymerase chain reaction
PIP2: phosphatidylinositol bisphosphate
PLCβ2: phospholipase C beta 2
qRT-PCR: quantitative real-time reverse transcription polymerase chain reaction
SEM: standard error of the mean
SOX2^+^: sex-determining region Y box 2
TNFα: tumor necrosis factor α
TNFR1: tumor necrosis factor 1
TNFR2: tumor necrosis factor 2
WAT: white adipose tissue
WT: wild-type
