# Supplementary figures and images for: Inflammation arising from obesity reduces taste bud abundance and inhibits renewal
Source: PLoS Biol. 2018 Mar 20;16(3):e2001959. doi: 10.1371/journal.pbio.2001959 (PMC5860696; doi:10.1371/journal.pbio.2001959)

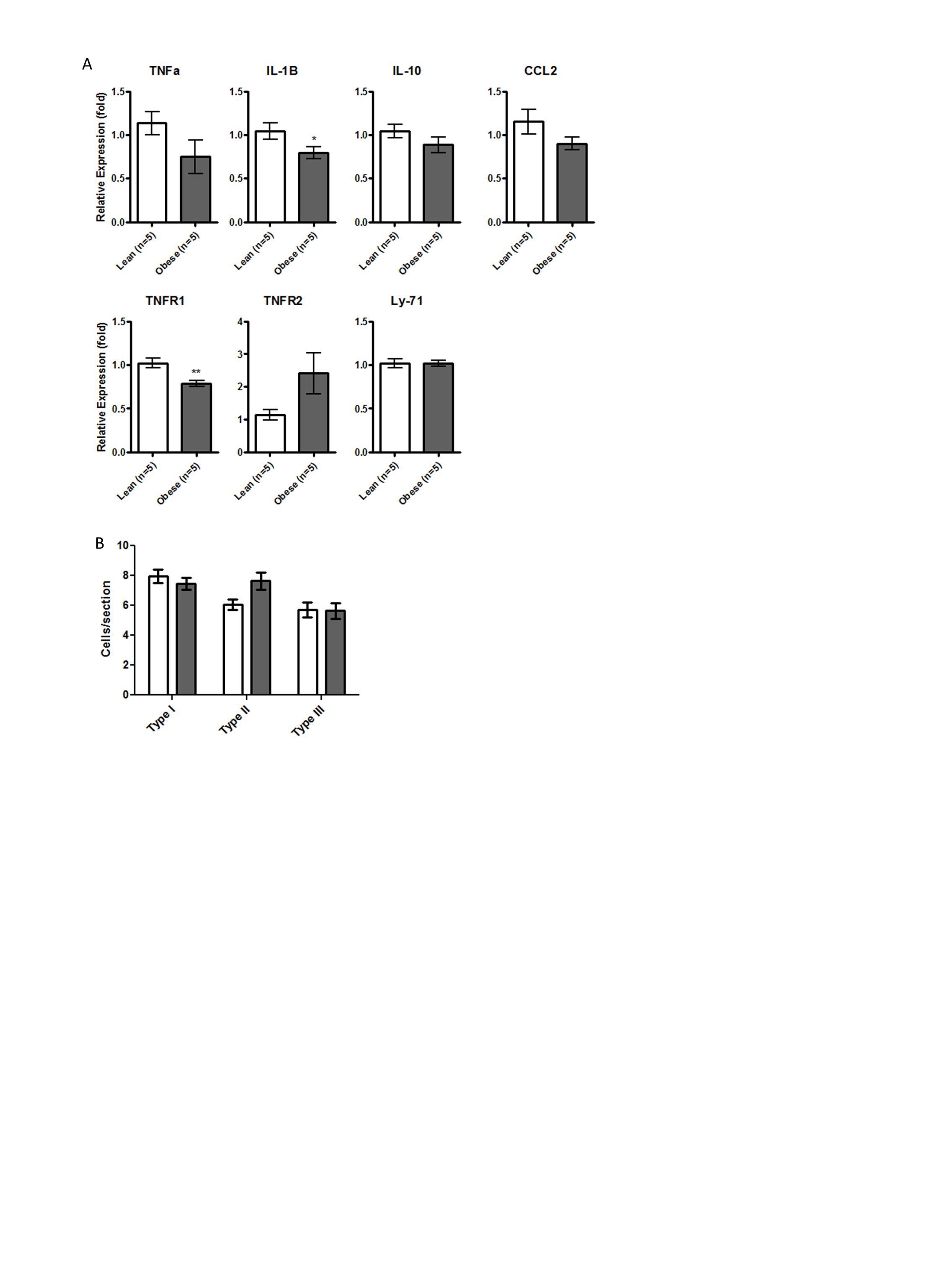

Supplement: S1 Fig — (A) qRT-PCR of nontaste epithelium reveals the majority of immune response from obesity arises from taste buds themselves (n = 5 each). Relative gene expression levels are shown (fold change). β-actin was used as the endogenous control gene for relative quantification. * = p < 0.05; ** = p < 0.01; *** = p < 0.005. Underlying data can be found in S1 Data. qRT-PCR, quantitative real-time reverse transcription polymerase chain reaction. (TIF) [file pbio.2001959.s001.tif]

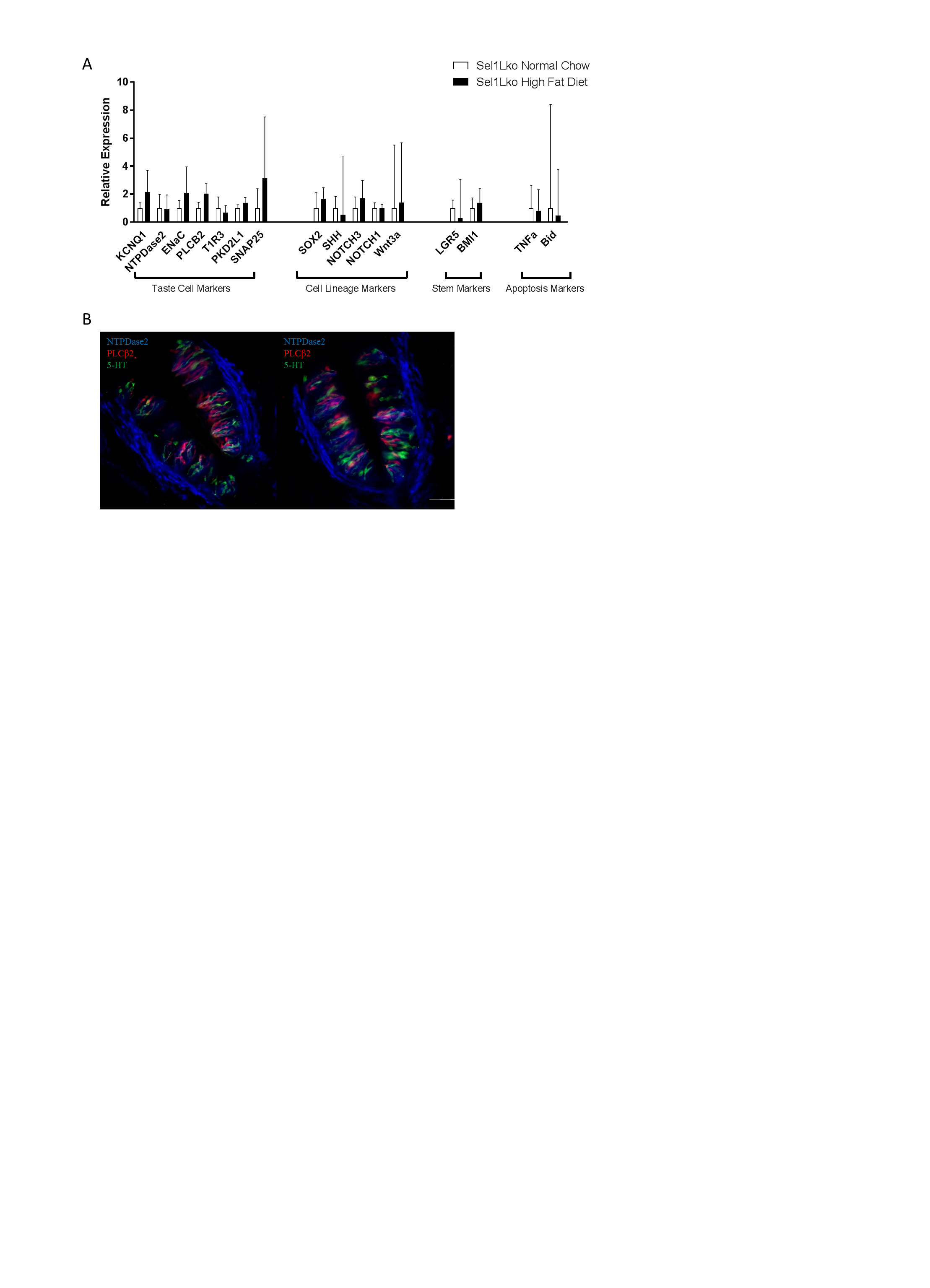

Supplement: S2 Fig — (A) Minimal change to markers of taste, cell lineage, or apoptosis in mice resistant to obesity. Relative gene expression levels are shown (fold change). β-actin was used as the endogenous control gene for relative quantification. (B) Representative images revealing no taste bud loss in obesity-resistant mice. Error bars represent SEM. * = p < 0.05; ** = p < 0.01; *** = p < 0.005. Scale bars, 50 μm. Underlying data can be found in S1 Data. SEM, standard error of the mean. (TIF) [file pbio.2001959.s002.tif]
