# Supplementary material for: Inflammation arising from obesity reduces taste bud abundance and inhibits renewal
Source: PLoS Biol. 2018 Mar 20;16(3):e2001959. doi: 10.1371/journal.pbio.2001959 (PMC5860696; doi:10.1371/journal.pbio.2001959)
Supplement: S1 Table — (DOCX) [file pbio.2001959.s004.docx]

| **Gene Name** | **GenBank Accession #** | **Forward Primer** | **Reverse Primer** |
| --- | --- | --- | --- |
| Taste Cell Markers: |  |  |  |
| NTPDase2 | NM_009849 | AGCTGGAGGATGCCACAGAG | GAGAGCAACCCAGGAGCTGA |
| PLCβ2 | NM_177568 | GAGCAAATCGCCAAGATGAT | CCTTGTCTGTGGTGACCTTG |
| T1R3 | NM_031872 | GAAGCATCCAGATGACTTCA | GGGAACAGAAGGACACTGAG |
| KCNQ1 | NM_008434 | GGAACATAGGGATGGGGAGT | GTTCCCTGATGGTCTCTGGA |
| Proliferation Markers: |  |  |  |
| LGR5 | NM_010195 | CCTACTCGAAGACTTACCCAGT | GCATTGGGGTGAATGATAGCA |
| β-Catenin | NM_007614 | GGCCTCTGATAAACGCTACTGTTG | ACGCAAAGGTGCATGATTTG |
| Ki67 | NM_001081117 | TCTGATGTTAGGTGTTTGAG | CACTTTTCTGGTAACTTCTTG |
| Inflammatory Markers: |  |  |  |
| TNF-α | NM_013693 | CCTCACACTCAGATCATCTTCTCA | TGGTTGTCTTTGAGATCCATGC |
| IL-6 | NM_031168 | TCATATCTTCAACCAAGAGGTA | CAGTGAGGAATGTCCACAAACTG |
| IL-1β | NM_008361 | GTAATGAAAGACGGCACACC | ATTAGAAACAGTCCAGCCCA |
| TNFR1 | NM_011609.4 | CCGGGAGAAGAGGGATAGCTT | TCGGACAGTCACTCACCAAGT |
| TNFR2 | NM_011610.3 | ACACCCTACAAACCGGAACC | AGCCTTCCTGTCATAGTATTCCT |
| IL-10 | NM_010548.2 | GCTCTTACTGACTGGCATGAG | CGCAGCTCTAGGAGCATGTG |
| Ly-71 | NM_010130.4 | TTGTACGTGCAACTCAGGACT | GATCCCAGAGTGTTGATGCAA |
| CCL2 | NM_011333.3 | TTAAAAACCTGGATCGGAACCAA | GCATTAGCTTCAGATTTACGGGT |
| Endogenous Control: |  |  |  |
| β-Actin | NM_007393 | CACCCTGTGCTGCTCACC | GCACGATTTCCCTCTCAG |
